# Geographic Distribution of Chagas Disease Vectors in Brazil Based on Ecological Niche Modeling

**DOI:** 10.1155/2012/705326

**Published:** 2012-02-27

**Authors:** Rodrigo Gurgel-Gonçalves, Cléber Galvão, Jane Costa, A. Townsend Peterson

**Affiliations:** ^1^Laboratório de Parasitologia Médica e Biologia de Vetores, Área de Patologia, Faculdade de Medicina, Universidade de Brasília, Campus Universitário Darcy Ribeiro, 70904-970 Brasília, DF, Brazil; ^2^Laboratório Nacional e Internacional de Referência em Taxonomia de Triatomíneos, Instituto Oswaldo Cruz, FIOCRUZ, 21045-900 Rio de Janeiro, RJ, Brazil; ^3^Laboratório de Biodiversidade Entomológica, Instituto Oswaldo Cruz, FIOCRUZ, 21045-900 Rio de Janeiro, RJ, Brazil; ^4^Biodiversity Institute, The University of Kansas, Lawrence, KS 66045-7593, USA

## Abstract

Although Brazil was declared free from Chagas disease transmission by the domestic vector *Triatoma infestans*, human acute cases are still being registered based on transmission by native triatomine species. For a better understanding of transmission risk, the geographic distribution of Brazilian triatomines was analyzed. Sixteen out of 62 Brazilian species that both occur in >20 municipalities and present synanthropic tendencies were modeled based on their ecological niches. *Panstrongylus geniculatus* and *P. megistus* showed broad ecological ranges, but most of the species sort out by the biome in which they are distributed: *Rhodnius pictipes* and *R. robustus* in the Amazon; *R. neglectus*, *Triatoma sordida*, and *T. costalimai* in the Cerrado; *R. nasutus*, *P. lutzi*, *T. brasiliensis*, *T. pseudomaculata*, *T. melanocephala*, and *T. petrocchiae* in the Caatinga; *T. rubrovaria* in the southern pampas; *T. tibiamaculata* and *T. vitticeps* in the Atlantic Forest. Although most occurrences were recorded in open areas (Cerrado and Caatinga), our results show that all environmental conditions in the country are favorable to one or more of the species analyzed, such that almost nowhere is Chagas transmission risk negligible.

## 1. Introduction

Chagas disease or American trypanosomiasis is a chronic and potentially fatal infection caused by the protozoan *Trypanosoma cruzi* [1]. Contamination of mucosa by feces of blood-sucking infected insects (Hemiptera, Reduviidae, Triatominae) is the most important way of transmission, althought transmission may also occur congenitally, by blood transfusion, from organ donors, and orally, via ingestion of food contaminated with *T. cruzi*. No vaccines, or effective antiparasitic treatments are available to cure the chronic phase of Chagas disease, so control of domiciliated vectors is the main strategy to prevent human infection [2–4]. Chagas disease, originally restricted to Latin America, is now becoming a global public health concern in nonendemic areas owing to human migrations to developed countries [5].

In Brazil, the Chagas disease national control program was implemented in 1975–1983, when *Triatoma infestans* infested domiciles of 700 municipalities in 12 Brazilian states [6]. At that time, 4.2% of the Brazilian population was estimated to be infected and around 100,000 new cases were recorded per year [7]. In 1991, Brazil joined the Southern Cone Initiative, an international consortium with the main objective of reducing vectorial transmission through insecticide spraying against *T. infestans*. After 10 years of effort, the project had a crucial impact on Chagas disease transmission in the Southern Cone countries, resulting in a 94% reduction of disease incidence [8]. Subsequent initiatives were also launched in the Andean region, Central America, and Amazonia [9]. The total prevalence of Chagas disease was reduced from >16 million to 8 million people, estimated in 2005 [10] and numbers of deaths were also reduced drastically [11]. In 2006, the Intergovernmental Initiative of Southern Cone, OMS, certified Brazil as free of vectorial transmission by *T. infestans* [12, 13]. In Brazil, the current estimate is that 1.9 million people are infected [10], much lower than the 6 million estimated in the 1980s [8]. 

The last Brazilian seroprevalence inquiry was carried out in 2001–2008, including 104,954 children, in which only 32 cases were detected, indicating significant reduction of transmission in recent years [14]. Nonetheless, acute cases of Chagas disease are presently being recorded in Brazil, mainly in the Amazon region [15]. In those cases, transmission involves either sylvatic vectors invading houses, food contamination or domestic/peridomestic populations of native triatomine species. The occurrence of those triatomines represents a great difficulty for the achievements via vectorial control [16–18].

Currently, in the subfamily Triatominae, 142 species are grouped in 18 genera and five tribes [19–24]. Studies of the geographic distribution of these species are crucial for understanding epidemiologic aspects of *Trypanosoma cruzi* transmission, and must be considered to orient control and monitoring of the disease. The study of the potential geographic distribution of important vector species is crucial for understanding geographic dimensions of risk transmission of the disease. In this context, ecologic niche modeling (ENM) is a tool that permits exploration of geographic and ecologic phenomena based on known occurrences of the species [25, 26]. ENM has been applied broadly to understanding aspects of Chagas disease transmission in the last decade, including characterization of the niches of vector species, and analysis of relationships between vector and reservoir distributions [27–37]. The objective of the present study is to analyze the geographic distributions of the triatomine species in Brazil and the factors related to their areas of occurrence. Ecologic aspects of the most important species are discussed, contributing to the knowledge of Chagas disease vectors in Brazil.

## 2. Material and Methods

### 2.1. Distribution Data

Distribution data of triatomine species in Brazil were obtained mainly from Lent and Wygodzinsky [38], Silveira and colleagues [6], and Carcavallo and colleagues [39]. New records were obtained from more recent studies [32, 33, 36, 37, 40–65]. We also obtained distributional data from species described after 1999 [22, 66–71]. Additionally, we analyzed triatomine records in the collections of Rodolfo Carcavallo and Herman Lent in the Laboratório Nacional e Internacional de Referência em Taxonomia de Triatomíneos, Instituto Oswaldo Cruz, FIOCRUZ. Recent information on the distribution of triatomines provided by Brazilian State Health Departments was also included.

### 2.2. Ecological Niche Models

#### 2.2.1. Input Data

We compiled 3563 records of triatomines in Brazil that could be referenced to geographic coordinates with a reasonable degree of confidence (i.e., with an uncertainty of ≤5 km, to a precision of ±0.01°). All records were georeferenced based on consultation of http://www.fallingrain.com/world/ and http://www.ibge.gov.br/. Eliminating duplicate records at this spatial resolution and removing a few records that presented obvious errors of georeferencing or identification, 3223 records remained, which documented occurrence of 62 triatomine species in the country. These data were organized in spreadsheets for analysis.

We set an occurrence data sample size criterion of 20 unique latitude-longitude points per species as a minimum to permit robust ENM development, based on previous analyses [72, 73] and extensive experience with such applications. This threshold left a total of 17 species: *Panstrongylus megistus*, *P. lutzi*, *P. geniculatus*, *Triatoma pseudomaculata*, *T. rubrovaria*, *T. sordida*, *T. tibiamaculata*, *T. petrocchiae*, *T. brasiliensis*, *T. melanocephala*, *T. costalimai*, *T. vitticeps*, *Psammolestes tertius*, *Rhodnius neglectus*, *R. nasutus*, *R. pictipes,* and *R. robustus*. In view of the exclusively sylvatic nature of the species *Ps. tertius* [63], we did not include this species in subsequent analyses. Barve and colleagues [74] recently recommended that niche and distribution models be calibrated across areas coinciding with the dispersal area (termed “**M**” by them) in the Biotic-Abiotic-Mobility (BAM) framework for understanding species geographic distributions [75], an assertion with which we concur amply. As essentially all of the species in this study have broad geographic distributions, we are comfortable with a relatively broad definition of **M**. However, a modification of the BAM framework for definition of calibration areas is based on considering the area that was actually sampled to produce the occurrence data for a study, which we can term **S**. Because both **M** and **S** determine the area that can possibly produce a presence record, albeit for very different reasons, and because our occurrence data came only from the extent of Brazil, we define our calibration area as **M** *∩* **S**, which in this case is the whole extent of Brazil. 

To characterize environmental variation across Brazil, we used two very different environmental datasets: multitemporal remotely sensed imagery and climatic data. The multitemporal (monthly) normalized difference vegetation index values (NDVI, a “greenness” index) were drawn from the Advanced Very High Resolution Radiometer satellite. These datasets have a native spatial resolution of ~1 km. The monthly nature of these greenness indices (April 1992–March 1993) effectively provides a detailed view of vegetation phenology across the country, which has proved quite useful and informative in recent analyses [76]. The years 1992-1993 coincide well with the temporal provenance of much of the occurrence data.

The second environmental dataset consisted of “bioclimatic” variables characterizing climates during 1950–2000 drawn from the WorldClim data archive [77]. To avoid the confounding effects of calibrating models in an overly dimensional environmental space [78], we chose only a subset of the 19 “bioclimatic” variables in the climatic data archive: annual mean temperature, mean diurnal range, maximum temperature of warmest month, minimum temperature of coldest month, annual precipitation, precipitation of wettest month, and precipitation of driest month. Both environmental datasets were resampled to 0.0417° spatial resolution for analysis, to match the approximate precision of the georeferencing of the occurrence data.

#### 2.2.2. Modeling Strategy and Methods

Different algorithms have different strengths and weaknesses in particular situations [79], which makes the choice of method an important consideration. In view of the particular characteristics of this study—that is, the area of interest is the area sampled, with no need for transfer of models over broader or other landscapes—Maxent appeared to be an excellent choice. Although recent comparative studies identifying Maxent as the “best” algorithm [80] are oversimplifications, our experience and analysis [81] indicate that it is indeed the ideal choice for this particular study. We used default parameters, except that we chose a random seed, with 5 replicate analyses based on bootstrap subsampling; we used the median output grids as the best hypothesis of potential range and imported them into ArcGIS 10 as floating point grids.

 For model results, for each combination of species with environmental dataset, we thresholded raw Maxent output independently. To emphasize the fact that omission error takes considerable precedence over commission error in niche modeling applications, we used a modified version of the least training presence thresholding approach [82] that takes into account the fact that some degree of error may exist in a dataset (otherwise, model predictions thresholded using this approach will be broadened artificially by error). Specifically, we estimated *E*, the expected amount of meaningful error in occurrence data [81] at 5%, and thresholded models at the suitability level that included 100 − *E* = 95% of the model calibration data to produce binary models summarizing likely presence and absence over the landscape. Finally, we combined the models from the two environmental datasets by multiplying them—this step had the effect of retaining areas as suitable only if they were judged as suitable by models based on both the AVHRR and climatic datasets. We note that because our initial hypothesis was that all of Brazil is available to each species for potential colonization, we took no specific steps to reduce model predictions from potential to actual distributional areas.

 Analyses were focused on the species diversity of broadly distributed (i.e., included within our initial list of well-sampled species) and human-disease-important triatomine species. As a result, we took the final, thresholded, and combined map for each of the 16 species listed above and summed them—given the binary nature of each of the maps, the sums yield a hypothesis of numbers of species present across Brazil. To provide a view of species' responses to environmental variation across Brazil, we plotted 1000 random points across the country, and assigned to each (1) the predicted presence or absence of each species and (2) the values of the first two principal components of the bioclimatic data set, which summarize much of the variation in climatic dimensions in a readily accessible and visualizable space of two dimensions.

## 3. Results

### 3.1. Diversity Patterns

Sixty-two of the 142 known triatomine species have been found in Brazil (Table 1). Thirty-nine out of 62 species (63%) occur only in Brazil. The state of Bahia, in northeastern Brazil, has the largest number of species (25 spp.), followed by Mato Grosso (18 spp.) in the central-west, Para and Tocantins (15 spp.) in the north, and Minas Gerais (15 spp.) in the Southeast. Rio Grande do Sul is the state with the largest number of species in southern Brazil (11 spp.). Acre and Amapá are the states with the lowest number of recorded species (Table 1).

**Figure 1:**
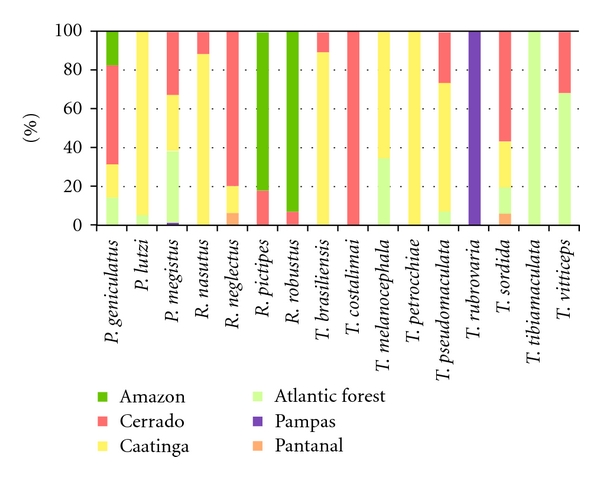
Relative occurrence of 16 species of triatomines across biomes, calculated based on proportions of known occurrences falling in each area.

 Grouping the 62 triatomine species by biome, we noted that the greatest number of species inhabits the Cerrado biome (*n* = 24; 39%), followed by the Amazon (*n* = 16; 26%), Caatinga (*n* = 15; 24%), and Atlantic Forest (*n* = 15; 24%). Fewer species were recorded in the Pantanal (*n* = 9; 15%) and Pampas (*n* = 8; 13%) biomes. The geographic distributions of some species coincide strongly with the distribution of particular biomes, while other species (e.g., *P. geniculatus* and *T. sordida*) occurred in at least four biomes (Figure 1). Most of these species occur in open areas in the cerrado and caatinga biomes, where the majority (70%) of occurrences were recorded.

### 3.2. Ecological Niche Models

**Figure 2:**
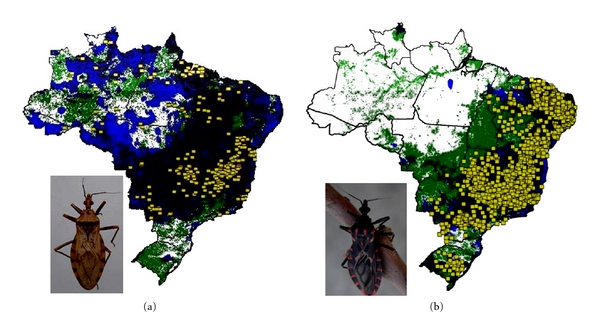
Ecological niche models projected as potential distributions for triatomine species with widespread distribution in Brazil. (a) *Panstrongylus geniculatus* and (b) *P. megistus*. Known occurrences of the species are shown as yellow squares, and the final consensus prediction is shown as black shading. Areas identified as suitable based on climatic grounds only are shown in blue, whereas areas identified as suitable based on normalized difference vegetation index (NDVI) only are shown in green.

**Figure 3:**
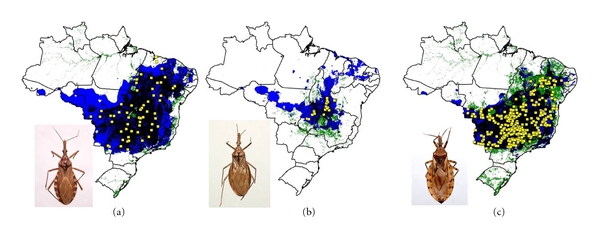
Ecological niche models projected as potential distributions for triatomine species in central Brazil. (a) *R. *neglectus, (b) *T. costalimai*, and (c) *T. sordida*.

**Figure 4:**
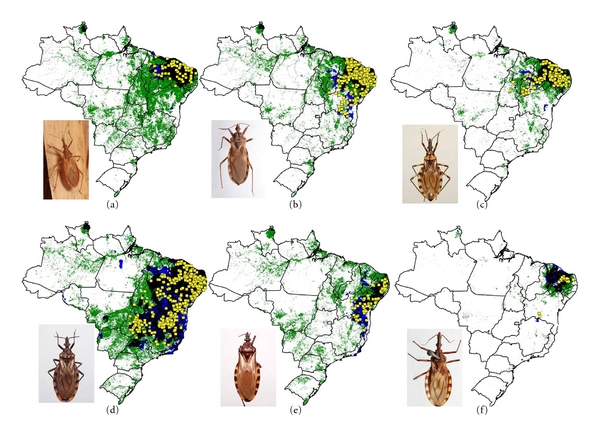
Ecological niche models projected as potential distributions for triatomine species in northeastern Brazil. (a) *R. nasutus,* (b) *P. lutzi, *(c) *T. brasiliensis, *(d)* T. pseudomaculata, *(e) *T. melanocephala, *and (f)* T. petrocchiae*.

**Figure 5:**
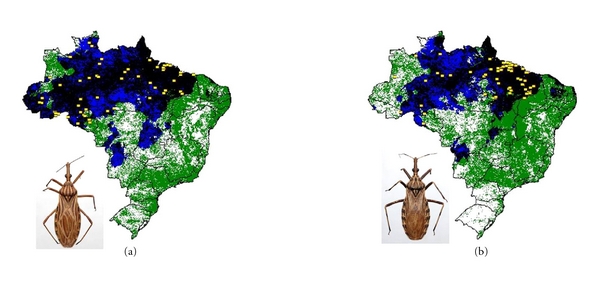
Ecological niche models projected as potential distributions for Amazonian triatomine species. (a) *R. robustus,* and (b) *R. pictipes*.

**Figure 6:**
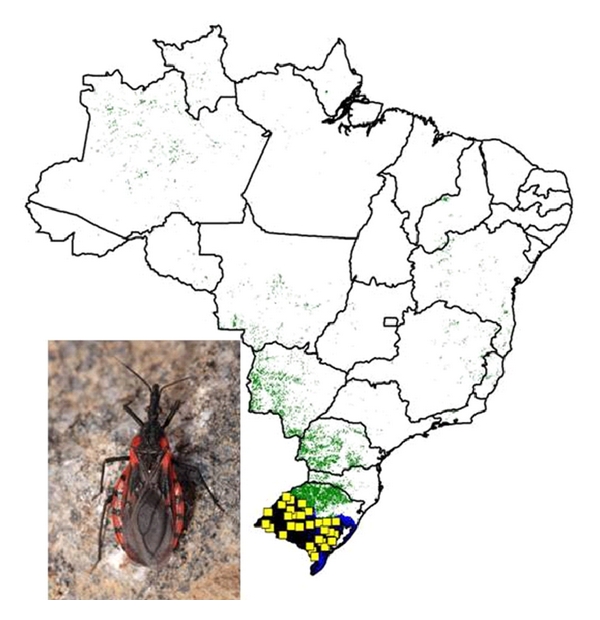
Ecological niche models projected as potential distribution for *T. rubrovaria*.

**Figure 7:**
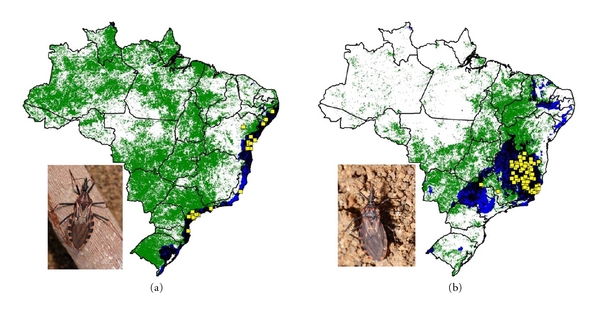
Ecological niche models projected as potential distributions for Atlantic Forest triatomine species. (a) *T. tibiamaculata* and (b) *T. vitticeps*.

We modeled the ecological niches of the 16 Brazilian triatomine species that are well sampled and present synanthropic tendencies. Of these species, *P. geniculatus* and *P. megistus* showed broad ecological and geographic distributions (Figure 2). The distributions of *R. neglectus*, *T. costalimai,* and *T. sordida* coincided with the Cerrado biome in central Brazil (Figure 3). On the other hand, distributions of *R. nasutus*, *P. lutzi*, *T. brasiliensis*, *T. pseudomaculata*, *T. melanocephala,* and *T. petrocchiae* coincided with the Caatinga of northeastern Brazil (Figure 4). We also noted higher probability of *R. robustus* and *R. pictipes* in northern Brazil (Figure 5), *T. rubrovaria* in the south (Figure 6), and *T. tibiamaculata* and *T. vitticeps* in the Atlantic Forest (Figure 7).

### 3.3. Factors That Determine Species' Distributions

**Figure 8:**
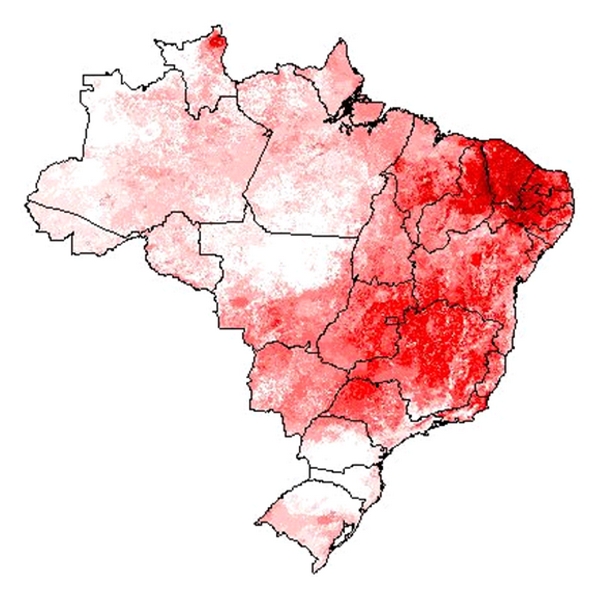
Triatomine species diversity map of Brazil, based on ecological niche models for 16 species of triatomine bugs. White areas have no species predicted as occurring, out of the 16 included in this analysis, while the darkest red areas have 13 species predicting as cooccurring.

As noted in 3.1 and 3.2, clear associations exist between species' ranges of occurrence and biomes. The overall map diversity among the 16 species modeled (Figure 8) indicates that the areas most favorable for the occurrence of these species are concentrated in the Cerrado and Caatinga, in the diagonal of open areas of eastern South America; other concentrations were in open areas of the Amazon, especially in the state of Roraima.

**Figure 9:**
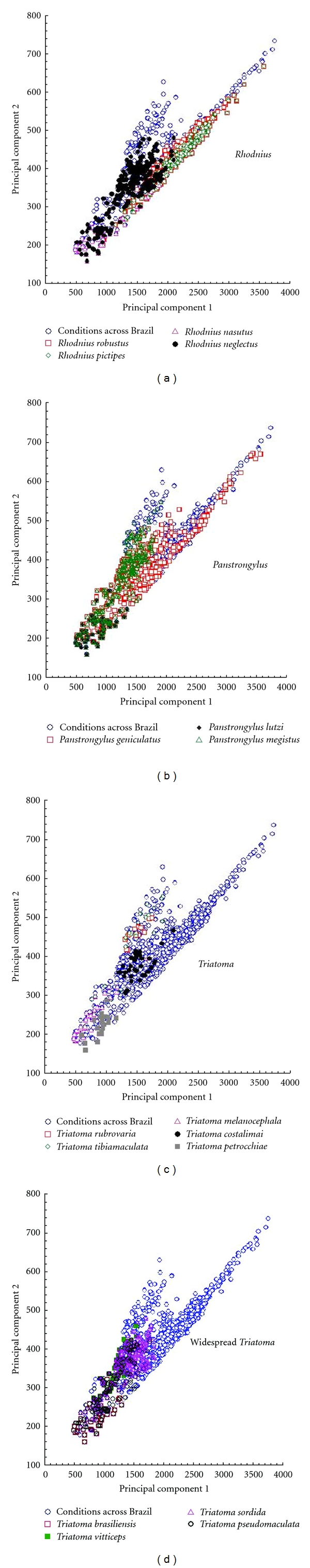
Distribution of 16 triatomine species in Brazil with respect to environmental variation summarized as the first two principal components summarizing variation in seven climatic dimensions. To provide a view of species' responses to environmental variation across Brazil, we plotted 1000 random points across the country, and assigned to each (1) the predicted presence or absence of each species and (2) the values of the first two principal components extracted from the bioclimatic data set.

The view of triatomine species' distributions across Brazilian environmental space shows the broad environmental diversity of the subfamily (Figure 9). Essentially all environmental conditions represented in the country are inhabited by one or more of the 16 species analyzed. Most of the species sort out by the region in which they are distributed: for example, *R. pictipes* and *R. robustus* in the Amazon, *Triatoma sordida* and *R. neglectus* in the Cerrado, *T. brasiliensis* and *P. lutzi* in the Caatinga, and *T. rubrovaria* in the far south. In sum, though, the view is of all of Brazil as having some suite of triatomine species present, such that almost nowhere is Chagas transmission risk negligible, although clearly some areas are at much higher risk than others.

## 4. Discussion

This study analyses the geographic distributions of the 62 species of triatomines in Brazil. We found that the great majority of species have a distribution restricted to fewer than 20 municipalities across Brazil. The analysis of the distribution of 16 synanthropic species broadly distributed in Brazil shows clear associations with Brazilian biomes, indicating the Cerrado and Caatinga as the ones presenting the highest diversity. The Brazilian states presenting the highest numbers of species are Bahia and Mato Grosso. The results suggest that essentially all environmental conditions represented across the country are inhabited by one or more of the 16 species analyzed.

*Panstrongylus megistus* was the species most broadly distributed throughout Brazil, as pointed out by Silveira [83]. The Atlantic Forest seems to represent the center of the range of *P. megistus* [84], although the species is also broadly distributed in humid areas of the Cerrado (“matas de galeria”) and Caatinga (forest remnants). *P. megistus* shows distinct levels of adaptation to domiciliary environments: in the south, it occurs mainly in sylvatic ecotopes [85] while in the southeast and in the northeast areas, where it is of epidemiologic importance, it occurs mainly in artificial ecotopes [84, 86, 87]. *P. megistus *seems to prefer hollow trees in arboreal habitats [88, 89] often associated with marsupials (*Didelphis *spp.), which are frequently infected by *T. cruzi*; this natural history explains the high levels of natural infection of *P. megistus* when compared to other triatomine vector species [90]. The broad geographic distribution, the acknowledged capacity to invade and colonize domiciles, and the high levels of *T. cruzi* infection indicate that *P. megistus* is the species of the greatest epidemiologic importance in Brazil after the control of *T. infestans*.

*P. geniculatus* is another triatomine species that is very broadly distributed in Brazil and the Americas, occurring in at least 16 countries [39, 48]. In sylvatic environments *P. geniculatus* preferentially inhabits armadillo nests (*Dasypus *spp.) [65, 88, 89] and domiciliary invasion by adults has been detected in several Brazilian states [6]; peridomiciliary colonies have also been found [91]. Nonetheless, its synanthropic behavior and vectorial competency are not as relevant as those of *P. megistus*. The bite of this triatomine is painful and causes allergic reactions in hosts, making blood meals in domestic environments difficult, consequently reducing the chances of colonization of artificial ecotopes [92]. The broad ranges of *P. geniculatus* and *P. megistus* could be facilitated naturally by mammals (marsupials and armadillos); future studies analyzing the geographic distribution of these triatomines in tandem with their potential hosts could clarify these possibilities.

In the Cerrado, the species most broadly distributed were *T. sordida* and *R. neglectus*. *T. sordida* occurs naturally under tree bark and also inhabits bird nests [93]. It is the most frequently captured species by entomological surveillance in Brazil [92, 94]. However, the risk of *T. cruzi* transmission by *T. sordida* is relatively low due either to the fact that it inhabits mainly the peridomiciliary ecotopes or to its ornithophilic behavior [88, 95]. According to Forattini [84] the areas of higher occurrence of *T. sordida* are the ones related to the agricultural activities in the past, what could explain its presence in areas that suffered ecologic impact due to significant loss of vegetation. It is important to stress that this process has been documented in the last decades in the Cerrado [96].

*Rhodnius neglectus* is widespread in the Cerrado, and plays an important role in the enzootic transmission of the *T. cruzi* [97]. Besides the invasion of adults in domiciles [54], the domiciliary colonization of *R. neglectus* has also been recorded in the states of Minas Gerais, São Paulo, and Goiás [98, 99]. *R. neglectus* occurs mainly in sylvatic environments, inhabiting different species of palm trees in Brazil [32, 55, 100]. The role of the peridomiciliary palm trees as a source of synanthropic *R. neglectus* must be evaluated in future studies.

*Triatoma sordida *and* R. neglectus* have also been recorded in other biomes (e.g., Caatinga and Pantanal). One of the possible explanations for this broad distribution would be passive transportation by birds. The dispersal could be facilitated by the fact that the eggs could be attached on the feathers, as suggested for the Rhodniini. Another possibility would be the transport of nymphs among the feathers as already shown for *T. sordida* [101]. One of the lines of evidence of this kind of dispersal is the coincidence between the distribution of the *R. neglectus* and birds like *Phacellodomus ruber *and *Pseudoseisura cristata *[32].

*Triatoma costalimai* seems to be endemic in the Cerrado. This species has been frequently captured in sylvatic environments (calcareous rocks), and also in peri- and intradomiciliar ecotopes in the municipalities of northeastern Goiás, with high rates of *T. cruzi* infection [102–104]. The geographic distribution of *T. costalimai* in the Cerrado remains poorly known, so research is needed to clarify its synanthropic behavior and vectorial capacity. In addition, ecological studies of other triatomine species in the Cerrado should be carried out to improve knowledge of the species occurring in this biome.

*Triatoma brasiliensis*, *T. pseudomaculata*, *P. lutzi*, and *R. nasutus* are broadly distributed in the Caatinga. *T. brasiliensis* is the most important vector species in northeastern Brazil [86, 105]. In sylvatic environments, it frequently inhabits rock crops in association with rodents (*Kerodon rupestris*) presenting significant levels of natural infection by *T. cruzi* [106]. The occurrence of *T. brasiliensis* may be associated with the distribution of the rock formations and “chapadas” in the northeast region of Brazil. This species is frequently found infesting houses in five states (MA, PI, CE, RN, and PB) [17, 107, 108]. It can also be found in very low numbers in the border areas of its distribution in the states of Tocantins and Pernambuco. The other four members of the *T. brasiliensis* species complex are either less synanthropic (*T. b. macromelasoma *and* T. juazeirensis*) or exclusively sylvatic (*T. melanica*) [21, 22]. *T. sherlocki*, recently included in this species complex, seems to be in the process of adaptation to the domiciliary environment [109].

*Triatoma pseudomaculata* occurs under bark of trees and in bird nests [88], presenting low percentages of natural infection by *T. cruzi* [86]. Although *T. pseudomaculata* can be found more frequently in peridomiciliary habitats, feeding generally on birds, in Ceará and Minas Gerais, full domiciliation has been observed [110, 111]. In some areas of the northeast, *T. pseudomaculata* has been the most common species after *T. brasiliensis*, so it must be kept under constant vigilance [86]. The presence of *T. pseudomaculata* in domiciliary environments has been linked to climate change and deforestation [111, 112]. One risk factor for domiciliation appears the passive transportation in wood for domestic use and construction of fences [92].

 The epidemiologic importance of *Panstrongylus lutzi* has been increasing in the last years. This species was the fifth most frequently found in captures across 12 Brazilian states in recent years [13, 105]. *P. lutzi* occurs in Caatinga and can be found inhabiting armadillo nests [113]; however, it has a much more eclectic diet in domestic environments and shows high levels of natural infection [45].

*Rhodnius nasutus* is found predominantly in Caatinga in the Northeast inhabiting palm trees of the species *Copernicia prunifera* [114, 115]. Nonetheless, *R. nasutus* may also occur in other palms and trees in the Caatinga [53, 116]. Although *R. nasutus* be considered as endemic to Caatinga, this species was registered in transitional areas with the Amazon forest (e.g., in the Maranhão Babaçu forests) and also with Cerrado [36].

 The geographic distribution of *Triatoma melanocephala* and *T. petrocchiae* was more restricted than the above-mentioned species. *Triatoma melanocephala* appears to occur in more humid areas than *T. petrochiae*. The natural habitats and food sources of these two species are little known [88], demanding further ecological study to clarify their vectorial potential in transmission of *T. cruzi* to humans.

*Triatoma tibiamaculata* and *T. vitticeps* present a geographic distribution more restricted to the Atlantic Forest. The former is frequently attracted by light but rarely colonizes houses [92]. This species was likely responsible for the contamination of the sugar cane juice that caused several cases of oral transmission recorded in the Santa Catarina [117]. In Bahia, *T. tibiamaculata* infected by *T. cruzi* has been found frequently in urban areas, mainly in the warmer months [118]. Adult specimens of *T. vitticeps* have been captured in rural areas of municipalities in Rio de Janeiro, Minas Gerais, and Espírito Santo [47, 62]. In contrast to *T. tibiamaculata*, *T. vitticeps* colonizes peridomiciliary areas, increasing risk of transmission of *T. cruzi* to humans [62, 92].

*Rhodnius robustus* and *R. pictipes* are broadly distributed in the Amazon region, as pointed out by Abad-Franch and Monteiro [119]. These species have several Amazonian palm trees species as their natural habitats, and present high percentages of natural infection by *T. cruzi* [55, 120]. Invasion of houses by adults, apparently related to artificial light sources, should favor transmission of *T. cruzi* to humans either by direct contact or by food contamination. This last possibility has been recorded frequently in the Amazon, where Chagas disease has been considered emergent [60, 121, 122]. Other possible scenarios of transmission in the Amazon would be of triatomine invasion of peridomiciliary ecotopes, as for *P. geniculatus* in Pará [91] and *T. maculata* in Roraima [44].

*Triatoma rubrovaria* presents a geographic distribution restricted to the Pampas biome in the south. In natural environments, the species can be found under rocks (sometimes very near to domiciles), where it feeds on several animals, including insects [123]. After the control of *T. infestans*, occurrence of this species has been increasing in Rio Grande do Sul [13, 124].

 The low occurrence of *T. infestans* detected in the present study is a further evidence for the effective reduction of this species in Brazil by the control programs [13, 125]. However, finding residual populations in Rio Grande do Sul and Bahia emphasizes the importance of constant entomological surveillance to avoid reinvasion by *T. infestans*.

## 5. Conclusions

Our results show that all environmental conditions in Brazil are favorable for one or more of the triatomine species analyzed, such that almost nowhere is Chagas transmission risk negligible. ENM presents a means of viewing species' ecology and biogeography across broad regions. It is by no means a substitute for detailed populational evaluations and natural history observations. Rather, used in tandem with local-scale studies, niche modeling provides complementary information. This study follows long years of previous exploration of these methodologies, and represents a step toward a complete distributional summary of the triatomines of Brazil.

## Figures and Tables

**Table 1:** Presence/absence matrix of triatomine species in Brazilian states. The rows represent the 62 triatomine species and columns the 27 Brazilian states. Presence records are represented by black cells in the matrix; white cells indicate that the species is probably absent in the state. The last row (labelled “Total”) indicates the total number of species recorded in each state, and the last columns indicate the total number of data points recorded and the biomes where each species occurs.

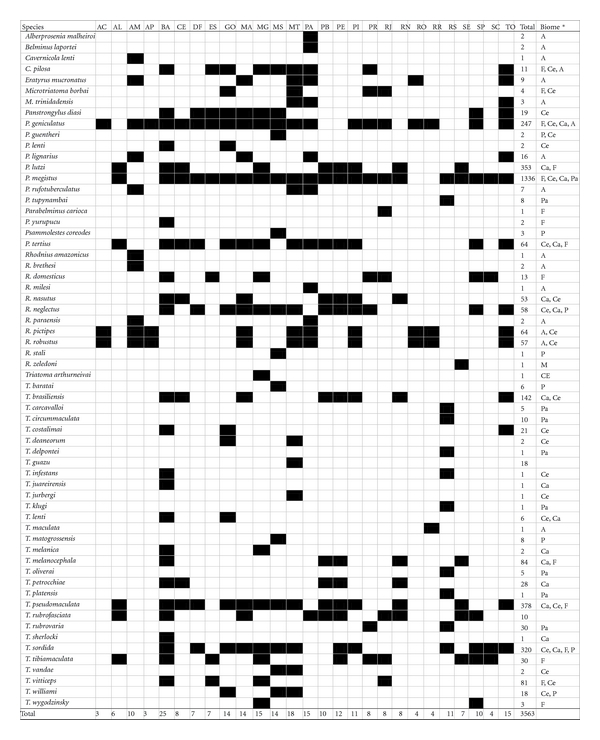

^∗^Biomes: Amazon (A), Cerrado (Ce), Caatinga (Ca), Pampas (Pa), Pantanal (P), and Atlantic Forest (F). ^∗∗^ *T. infestans* is a domestic species, nonendemic in Brazil. After control with insecticides this species is restricted to residual foci in two states. ^∗∗∗^ *T. rubrofasciata* is a cosmopolitan species. In Brazil, its presence was detected in all the major harbors. Brazilian state abbreviations: AC: Acre, AL: Alagoas, AM: Amazonas, AP: Amapá, BA: Bahia, CE: Ceará, DF: Distrito Federal, ES: Espírito Santo, GO: Goiás, MA: Maranhão, MG: Minas Gerais, MS: Mato Grosso do Sul, MT: Mato Grosso, PA: Pará, PB: Paraíba, PE: Pernambuco, PI: Piauí, PR: Paraná, RJ: Rio de Janeiro, RN: Rio Grande do Norte, RO: Rondônia, RR: Roraima, RS: Rio Grande do Sul, SE: Sergipe, SP: São Paulo, SC: Santa Catarina, and TO: Tocantins.
